# BiVO_4_-rGO with a novel structure on steel fabric used as high-performance photocatalysts

**DOI:** 10.1038/s41598-017-07342-1

**Published:** 2017-08-11

**Authors:** Dong Fang, Xiujuan Li, Hui Liu, Weilin Xu, Ming Jiang, Wenbin Li, Xin Fan

**Affiliations:** 1 Key Lab of Green Processing and Functional Textiles of New Textile Materials Ministry of Education, College of Material Science and Engineering, Wuhan Textile University, Wuhan, 410000 P. R. China; 2 School of Metallurgy and Environment, Central South University, Changsha, 410083 P. R. China; 3 College of Materials Science and Engineering, Guilin University of Technology, Guilin, 541004 P. R. China

## Abstract

A high-performance and novel photocatalyst of BiVO_4_-reduced Graphene Oxide (BiVO_4_-rGO) nanocomposite was prepared by a facile hydrothermal method. The photocatalyst was characterized by X-ray diffraction, X-ray photoelectron spectroscopy, scanning electron microscopy, transmission electronic microscopy, UV-Vis diffusion reflectance spectroscopy, photoluminescence spectroscopy and UV-Vis adsorption spectroscopy, respectively. The visible-light photocatalytic activity was evaluated by oxidation of methyl orange (MO) under simulated sunlight irradiation. The results show that the BiVO_4_-rGO nanocomposites exhibit enhanced photocatalytic performance for the degradation of MO with a maximum removal rate of 98.95% under visible light irradiation as compared with pure BiVO_4_ (57.55%) due to the increased light absorption intensity and the degradation of electron-hole pair recombination in BiVO_4_ with the introduction of the rGO.

## Introduction

Due to the rapid urbanization and industrialization, water pollutions has received increased attention, which presents a challenge to environmental governance^1^. Many methods are available for removing organic dyes from wastewater, including physical-^2^, biological-^3^, electrochemical-^4^, and oxidation- technology^5^. Among them, the advanced oxidation technology, especially the photocatalysis method, has become one of the most important techniques for the degradation of organic contaminants in wastewater^6–13^.

Monoclinic bismuth vanadate (BiVO_4_) has been widely used as a photocalalyst dye treatment under visible light irradiation^14^. The advantages of the compound include a narrow band gap for visible light absorption, abundant availability, low cost and good stability^15^. It is known that the photocatalytic properties of the material greatly depend on its the structure and morphology^16–18^. There are three main crystal structures for BiVO_4_: monoclinic scheelite, tetragonal zircon, and tetragonal scheelite^19^. Among them, the monoclinic BiVO_4_ is an n-type semiconductor with a narrow band gap of about 2.4 eV and it has an excellent photocatalytic activity in the visible range for the degradation of organic pollutants because of its appropriate band gap for solar energy conversion^20^. However, the low photocatalytic activity of pure BiVO_4_ has limited its further use in practical applications due to its poor adsorptive performance and migration difficulty of photo-generated electron-hole pairs^21, 22^. To overcome this problem, many efforts have been made to enhance the activity of BiVO_4_-based photocatalysts. Element dopants added to BiVO_4_ to increase the donor density and carrier mobility^23^ and BiVO_4_-based composites including homo/hetero-junction construction and co-catalyst loading metal oxide compounds have been investigated^24–27^. These results have shown that the BiVO_4_-based composites favor the separation of photo-induced electron-hole pairs and result in enhanced photocatalytic activity in the visible range^28^.

Recently, two-dimensional (2-D) graphene has increasingly attracted attention due to its fascinating physical properties including quantum electronic transport, extremely high mobility, high elasticity, and electromechanical modulation^29, 30^. Graphene oxide has a similar structure as grapheme and the only difference is that the surface and edges of the tgraphene oxide carbon skeleton are modified by oxygen-containing groups^31^ (such as -CO-, -OH-, -COOH, C-O-C). There are experiment interactions between the oxygenic functional groups of graphene oxide and different materials by a non-covalent bond, a covalent bond, or an ionic interaction mode, which can easily result in functionalized mixtures and composites with extraordinary properties easily. In recent years, graphene-based nano-materials have been utilized as photocatalysts to enhance the photocatalytic efficiencies because the such a supporting matrix with excellent electric conductivity and a super-high surface area make excellent contact with water or target pollutants to provide plenty of reactive sites^32–37^.

In this work, we present a simple hydrothermal method to prepare BiVO_4_-rGO composites using graphene oxide (GO) and Bi(NO_3_)_3·_5H_2_O as starting materials. The synthesis processes of BiVO_4_ nanowires and BiVO_4_-rGO nanosheets are presented in detail. The photocatalytic activities of BiVO_4_ and BiVO_4_-rGO are evaluated for the degradation of MO. The results indicate a superior photocatalytic performance of the BiVO_4_-rGO composite under simulated sunlight conditions.

## Results and Discussion

**Figure 1:**
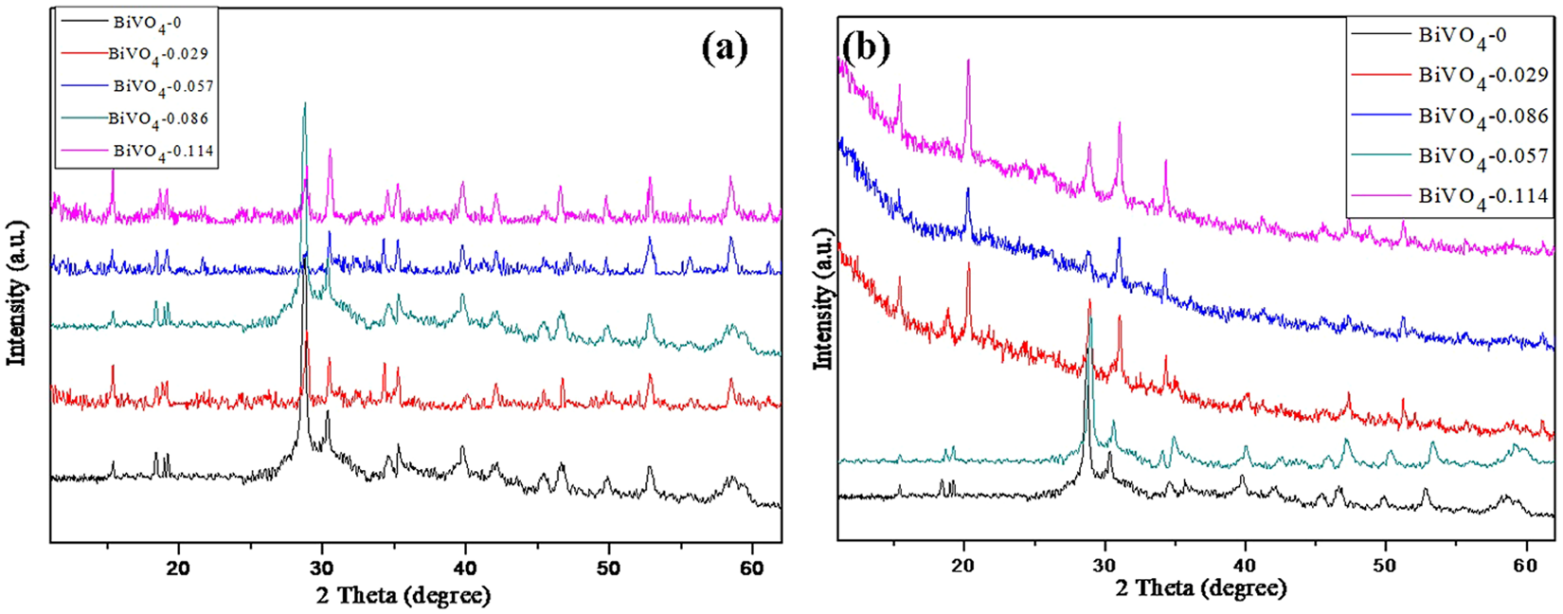
(**a**) XRD patterns of pristine BiVO_4_ and the BiVO_4_-rGO nanocomposites, (**b**) Raman spectra of the BiVO_4_, BiVO_4_-0.057 composite, and pure graphene oxide.

The crystallographic structure and phase purity of the as-obtained samples are first examined by powder X-ray diffraction (XRD) analysis (Fig. 1a). All the diffraction peaks can be indexed as the body-centered monoclinic phase of BiVO_4_ with lattice constants of *a* = 5.195 Å, *b* = 11.70 Å and *c* = 5.092 Å (JCPDS card no. 14-0688)^38–40^. The XRD patterns are similar for the BiVO_4_- rGO composites and the BiVO_4_. An increase in the content of GO results in no obvious changes in the XRD patterns of the samples, suggesting that the introduction of GO has little influence on the crystalline structure of BiVO_4._ The Raman spectra at room temperature under green laser excitation (532 nm) are shown in Fig. 1(b). The main Raman peaks of monoclinic BiVO_4_ are observed around 210, 325, 366, 707 and 827 cm^−1^, which are consistent with typical vibrational bands of monoclinic BiVO_4_^41, 42^. The dominating peak at 827 cm^−1^ and the inconspicuous peak at 707 cm^−1^ are assigned to the symmetric and antisymmetric V-O stretching mode, respectively. The Peak centered at 366 and 325 cm^−1^ is attributed to the typical symmetric and antisymmetric bending modes of the vanadate anion, respectively. The GO exhibits Raman shifts at 1591 and 1355 cm^−1^, corresponding to the G- and D-bands, respectively. As for the BiVO_4_-0.057 nanocomposites, aside from the distinctive peaks assigned to BiVO_4_, the G- and D-bands of rGO are located at 1588 and 1350 cm^−1^, respectively, indicating shifts toward lower wavenumbers as compared to GO^43, 44^.

**Figure 2:**
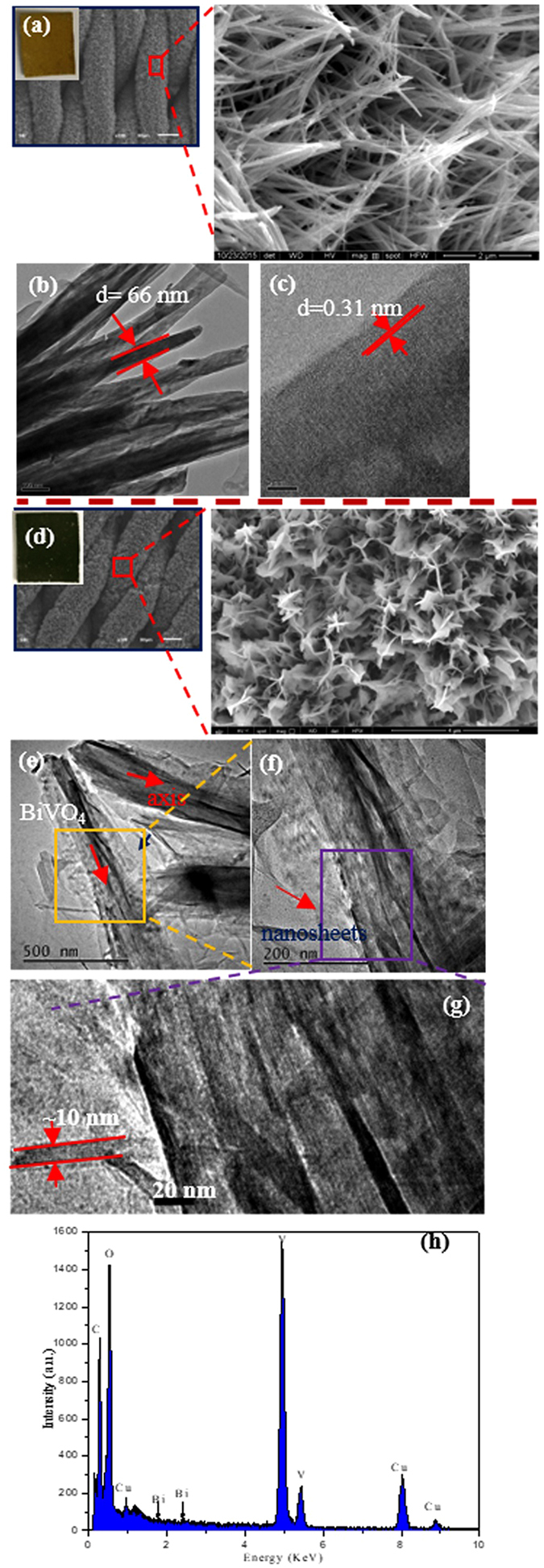
(**a**) SEM, (**B**) and (**C**) low- and high-magnification TEM images of the pure BiVO_4_, (**d**) SEM, (**e**) and (**f**) low- and high-magnification TEM images of the BiVO_4_-0.057 nanocomposites. Inset in (**a**) and (**d**) is the corresponding digital picture of sample, (**h**) EDS spectrum of BiVO_4_-0.057 nanocomposite.

Figure 2 present typical scanning electron microscopy (SEM) and transmission electron microscopy (TEM) images of the BiVO_4_ and BiVO_4_-0.057 products. As shown in Fig. 2a, it can be seen that the as-synthesized BiVO_4_ has a nanowire structure. The TEM image presented in Fig. 2b reveals that the diameter of BiVO_4_ is about 66 nm and that the interplanar distance of the (121) plane of the monoclinic BiVO_4_ is 0.31 nm (Fig. 2c)^45^. Interestingly, after BiVO_4_ was coupled with rGO, the morphology of the nanowires disappeared completely and sheets-like structures appeared, as shown in Fig. 2d. In the TEM images (Fig. 2e–g), there is a central BiVO_4_ nanowire axis, which is covered by BiVO_4_/rGO nanosheets. The thickness of the nanosheets is about 10 nm. The functional groups such as hydroxyl, carboxyl, and carbonyl groups of the GO may provide the reaction sites for the nucleation and growth of the BiVO_4_-0.057 nanosheets^46^. In the TEM-EDS image of the BiVO_4_-0.057 nanosheets is tested and presented (Fig. 2h), from which Bi, V, O and C signals are clearly observed.

**Figure 3:**
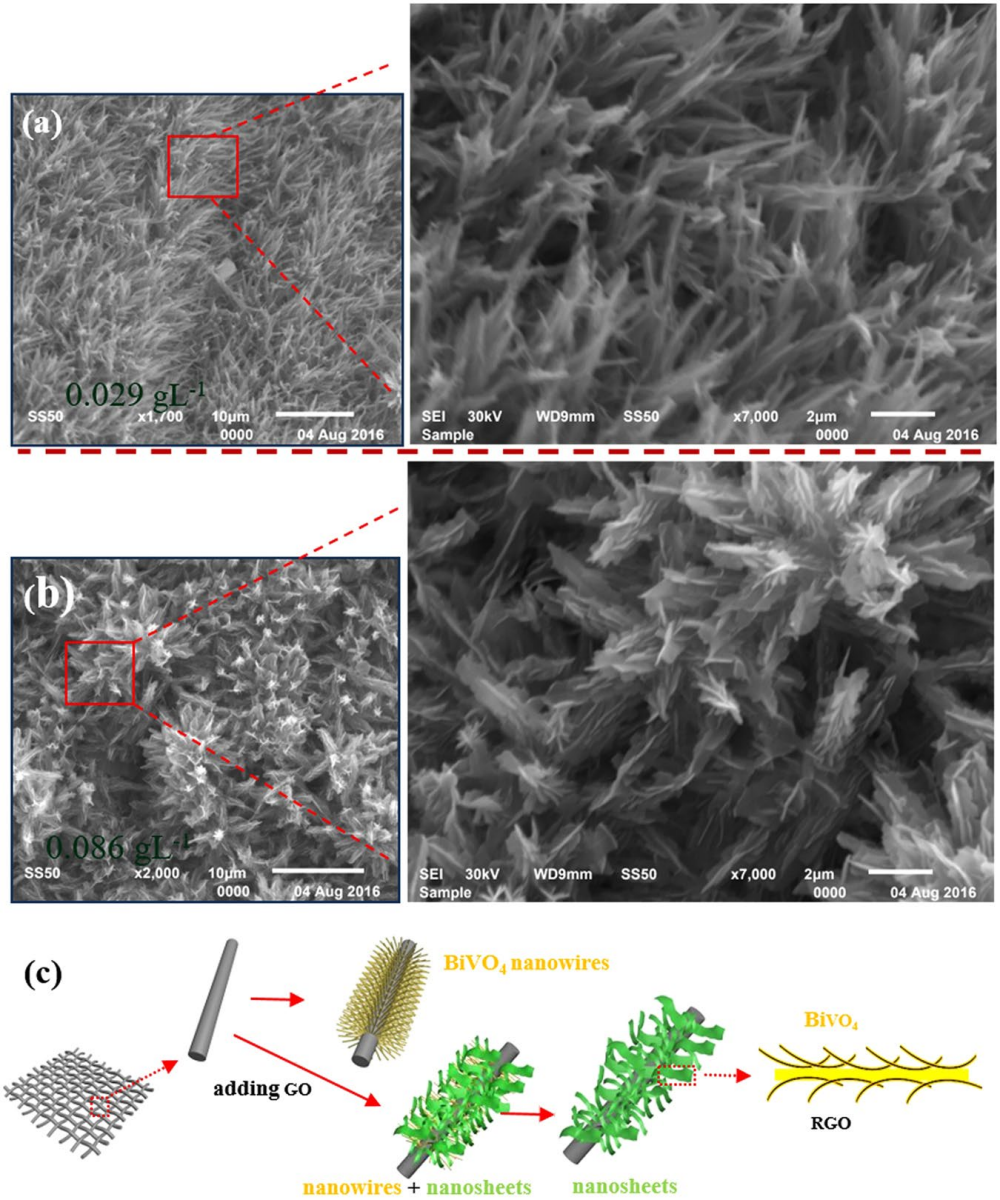
(**a**) and (**b**) SEM images of BiVO_4_-0.029 and BiVO_4_-0.086; (**c**) schematic illustration of the synthesis procedure of the BiVO_4_ nanowires and BiVO_4_-rGO nanosheets.

For a comparison, the morphological structures of the BiVO_4_-rGO nanocomposites with different synthesis conditions are characterized by the SEM technique(Fig. 3). When the concentration of GO is 0.029 gL^−1^, the images show that part of the nanowires are covered by nanosheets (Fig. 3(a)). When the GO amount is increased to 0.057 gL^−1^ (Fig. 2c), the nanowires disappear completely and 500-nm wide exhibit nanosheet structures are formed. With a further increase in the GO to 0.086 gL^−1^ (Fig. 3(b)), the nanosheet structure morphology is retained. Figure 3(c) presents the formation diagram of the BiVO_4_ nanowires and the BiVO_4_-rGO nanosheets process. Initially, the nanowire-like BiVO_4_ is obtained on a Ti fabric under hydrothermal condition. When the rGO is incorporated, a portion of the nanowires are covered by nanosheets. As the rGO amount is further increased, more nanosheets are formed on the nanowires. In the detailed structure of the nanosheets, the rGO nanosheets are covered by BiVO_4_, i.e., a sandwich structure is formed with rGO in the central part.

**Figure 4:**
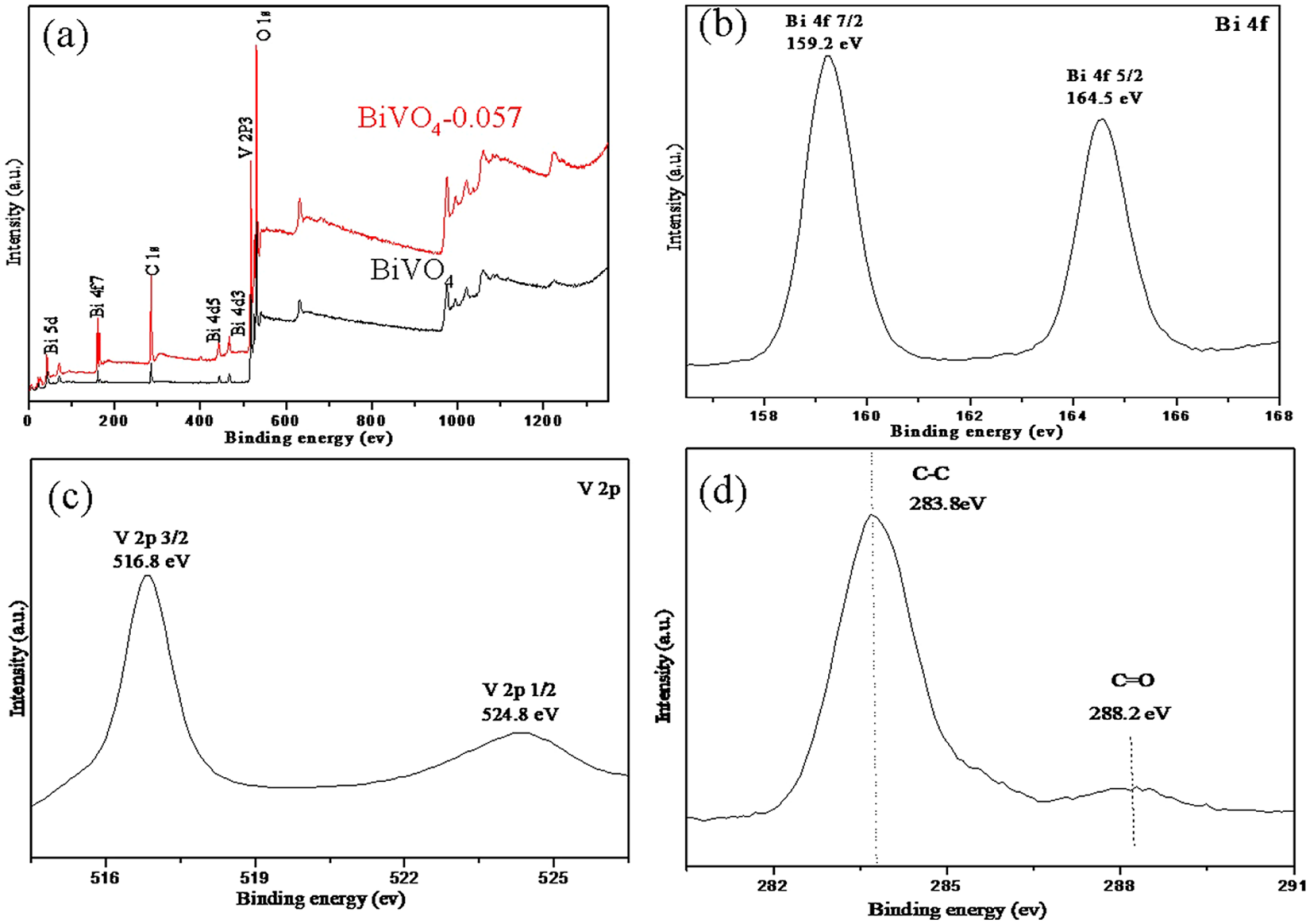
(**a**) Survey XPS spectra for BiVO_4_ and BiVO_4_-0.057, (**b**) Bi 4 f, (**c**) V 2p and (**d**) C 1 s for BiVO_4_-0.057.

The surface chemical composition and the chemical states of BiVO_4_ and BiVO_4_-0.057 were analyzed by X-ray photoelectron spectroscopy(XPS) (Fig. 4a). Seven obvious peaks corresponding to Bi5d, Bi4f, C1s, Bi4d^5/2^, Bi4d^3/2^, O1s and V2p^3/2^ are detected in both samples. Figure 4b–d shows the XPS spectrum of Bi(b),V(c) and C(d) in the BiVO_4_-0.057 composite, respectively. XPS signals of Bi 4 f with binding energies at 164.5 eV (Bi 4f^7/2^) and 159.2 eV (Bi 4f^5/2^) are detected (Fig. 4b), which confirm that the Bi species exist as Bi^3+^ ^47–51^. The signal of V 2p^1/2^ and V 2p^3/2^ is located at 524.8 and 516.8 eV, respectively (Fig. 4c), indicating that the V species are in the state of V^5+^ ^52, 53^. Thus, the electron couples of Bi^3+^ and V^5+^ coexist in the orthorhombic BiVO_4_ structures, where the total atomic ratio of the Bi and V elements is about 1:1, corresponding to the molecular formula of BiVO_4_. In the high resolution spectrum of C 1 s (Fig. 4d), carbons in the form of sp^2^ bonds (284.6 eV) are dominated and oxygen-containing functional group is also observed at 288.6 eV (C = O), which may represent the absorption of atmospheric CO_2_^54, 55^.

**Figure 5:**
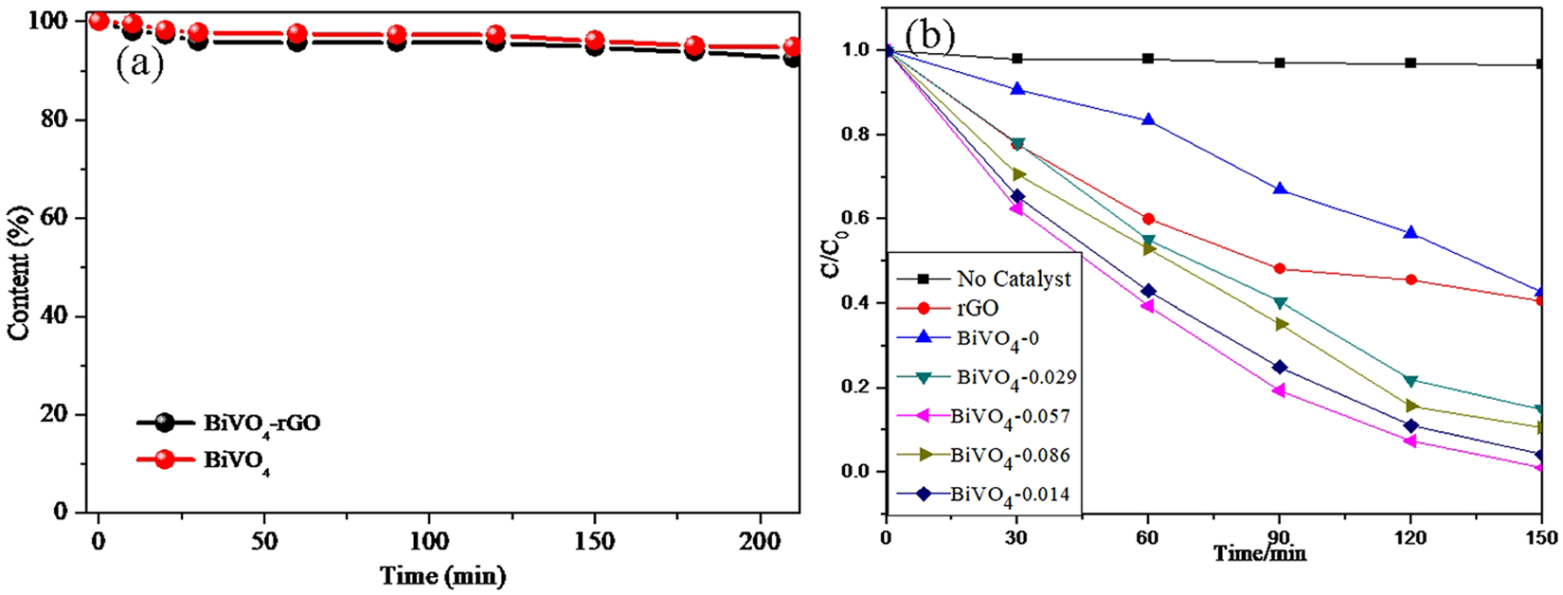
(**a**) The adsorption removal of MO in the dark by BiVO_4_-rGO nanocomposite arrays (black) and BiVO_4_ nanowire arrays (red), (**b**) Time-online photocatalytic performance of BiVO_4_ and BiVO_4_-rGO nanocomposite photocatalysts with different concentration of GO for the degradation of MO under simulated sunlight.

The normalized temporal concentration changes (C/C_0_) of MO during the photocatalytic process are proportional to the normalized maximum absorbance (A/A_0_), which can be derived from the change of the MO absorption profile at a given time interval. Figure 5a shows that the adsorption-desorption equilibrium attained in 210 minutes and the adsorption capacity were 7.30% and 5.10% of the MO for BiVO_4_-rGO nanocomposite arrays (black) and the BiVO_4_ nanowire arrays (red), respectively. As can be seen in Fig. 5b, the degradation of the MO solution exhibited a small decrease without the photocatalyst under visible-light irradiation, this decrease was 9.4% for 150 min of irradiation, indicating that the MO was stable. The photocatalytic performance of the BiVO_4_-rGO composites is dependent on the proportion of rGO in the composite. Under simulated sunlight irradiation for 150 min, pure rGO and BiVO_4_ exhibit 59.50% and 57.55% degradation efficiency for MO, respectively. When rGO is introduced into BiVO_4_, the removal rate is increased to 85.03% for BiVO_4_-0.029, and reaches a maximum value of 98.95% for BiVO_4_-0.057, while the removal rate is 89.37% and 88% for BiVO_4_-0.086 and BiVO_4_-0.114, respectively. The BiVO_4_-rGO composites exhibit a slightly lower activity, which is still significantly higher than that of the pure BiVO_4_ sample. It is known that during photocatalysis, the light absorption and the charge transportation and separation are crucial factors; these energy levels are beneficial for the transfer of photo-induced electrons from the BiVO_4_ conduction band to the rGO, which can efficiently separate the photo-induced electrons efficiently and hinder the charge recombination in the electron-transfer processes^56^, thus enhancing the photocatalytic performance. However, when the rGO content is further increased above its optimum value, the photocatalytic performance deteriorates. This is ascribed to the following reasons: (i) rGO may absorb some visible light and thus cause a light harvesting competition between BiVO_4_ and rGO with the increase of the rGO content, which leads to the decrease in the photocatalytic performance^57, 58^; (ii) the excessive rGO can act as a kind of recombination center instead of providing an electron pathway and promoting the recombination of electron-hole pairs in the rGO^59^.

**Figure 6:**
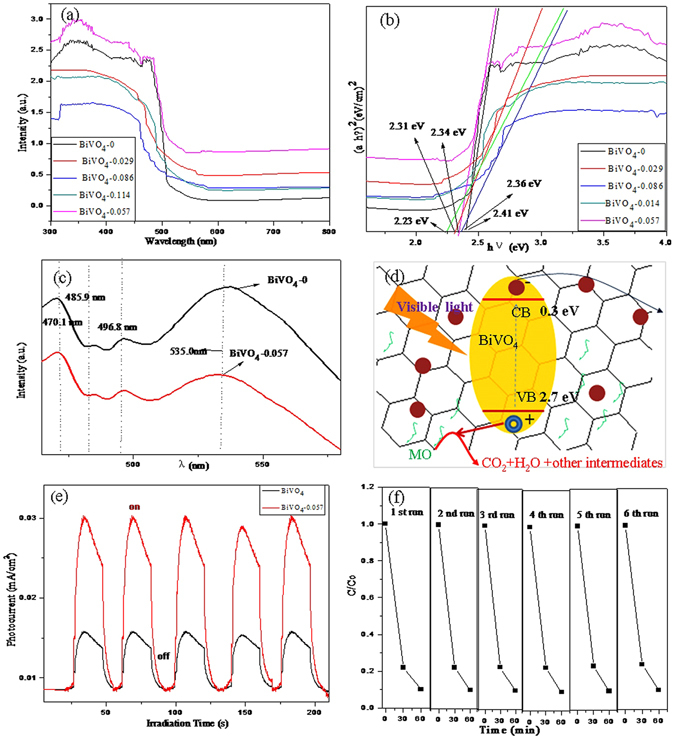
(**a**) and (**c**) UV-vis diffuse reflectance and photoluminescence spectral of bare BiVO_4_ and BiVO_4_-rGO nanocomposites, (**b**) plots of (*αhν*)^2^ versus photon energy (*hν*) of BiVO_4_ and BiVO_4_-0.057 nanocomposites, (**d**) schematic diagram for illuminating the charge behavior at the interface of BiVO_4_ and rGO, (**e**) Photocurrent densities of bare BiVO_4_ nanowire arrays (black) and BiVO_4_-rGO nanocomposite (red) under simulated sunlight, (**f**) 6 cycles of the photocatalytic degradation of MO using BiVO_4_-rGO as the photocatalyst under visible-light irradiation for 60 min.

UV-vis spectra are used to characterize the optical properties of the samples (Fig. 6a). According to the spectra, all the samples express absorbance in the visible regions. The pure BiVO_4_ exhibits an absorption edge at around 515.41 nm, showing a good visible light response. Accompanied by the introduction of rGO, the obtained BiVO_4_-0.057 composite exhibits an absorption edge at around 529.48 nm and a broader visible light absorption in the range of 500–700 nm. These results further confirm that the existence of rGO in the BiVO_4_-rGO composite greatly improves the visible-light absorption properties of the composite. Furthermore, the optical bandgap energy (Eg) of the obtained samples can be estimated from the formula (αhν) = A(hν-Eg)^n/2^ ^60, 61^, where α is the absorption coefficient, h is Planck’s constant, A is a constant, ν is the light frequency, and n = 1 and 4 for direct and indirect band gap materials, respectively. The optical transition of BiVO_4_ is direct and the value of n is thus 1. As shown in Fig. 6b, the approximated band gaps of pure BiVO_4_ and BiVO_4_-0.057 are 2.41 and 2.34 eV, respectively. The narrowing of the band gap can be ascribed to the chemical bonding between BiVO_4_ and the specific sites of GO during the hydrothermal treatment. This indicates that the BiVO_4_-rGO nanocomposites can be photoexcited to generate more electron-hole pairs under visible-light irradiation, which can result in a higher photocatalytic performance.

In addition, the photoluminescence (PL) spectrum is regarded as a significant emission signal of carrier recombination. The transfer property of the photogenerated carriers (electron-hole pairs) can be evaluated by this method. Usually, a weaker PL intensity indicates a stronger ability for the separation of photo-generated carriers^62^. Figure 6c shows the PL emission spectra of pure BiVO_4_ and the BiVO_4_-0.057 photocatalysts monitored at an excitation wavelength of 320 nm. The peak at ~535 nm corresponds to the recombination of the hole formed in the O 2p band and the electron in the V 3d band^63^, corresponding to the near band edge emission (NBE) of BiVO_4_^64^. A decrease in PL intensity is clearly evident for the BiVO_4_-0.057 due to the effective separation of electron-hole pairs. The photo-generated electrons in the excited BiVO_4_ are transferred to the rGO nanosheets immediately after the photo-production, separating the photo-generated electrons and holes and inhibiting their recombination (Fig. 6d). This may be the reason that the BiVO_4_-rGO sample exhibits an enhanced photocatalytic efficiency under visible light irradiation.

Figure 6e illustrates the photocurrent responses of the BiVO_4_-rGO as photoelectrode under intermittent illumination by simulated sunlight and compared with that of the bare BiVO_4_ nanowire arrays. The photocurrent density is much higher for the BiVO_4_-rGO nanocomposite arrays than for the BiVO_4_ nanowire arrays, suggesting that the charge carriers that are photogenerated for the BiVO_4_-rGO persist than those for the BiVO_4_ nanowire arrays. This is not surprising because the photo-responsive rGO contributes to the photocurrent. Further, the rGO possesses an enhanced charge mobility compared with the BiVO_4_ nanowire arrays.

Figure 6f shows the results of five successive runs for the photo-degradation of MO for the BiVO_4_-rGO composite photocatalyst under the same experimental conditions. There is no apparent loss of photoactivity after six consecutive photo-degradation cycles. Therefore, the BiVO_4_-rGO composites possess excellent stability and are not prone to be suffer from photo-corrosion during the degradation process.

## Conclusions

In summary, a novel BiVO_4_-rGO photocatalyst was successfully synthesized via a simple one-step hydrothermal method. Based on the narrow band gap (2.34 eV) and the relatively low PL intensity, the added rGO can effectively suppress the complex of light-generated electron-hole and increase the separation efficiency of photon-generated carrier, thereby enhancing the catalytic activity of the composite photocatalyst. The synthesized composite photocatalysts showed much higher photocatalytic activity than that of pure BiVO_4_ with regarding to MO degradation under visible light. The present recoverable BiVO_4_-rGO composite photocatalysts can be regards as one of the ideal photocatalysts for the various potential applications.

## Experimental Section

### Synthesis of a uniform BiVO_4_ nanowires

All reagents were of analytical grade and used as received without further purification. In a typical procedure, NH_4_VO_3_, oxalic acid, hexamethylenetetramine and Bi (NO_3_)_3_ 5H_2_O (the molar ratio 30:60:6:1) were dissolved into deionized water under ultrasonication for 1 h at room temperature. The dark blue mixture solution and a piece of pretreated Ti fabric, which has been rinsed with pure ethanol and deionized water for 1 h, were transferred into an autoclave, and then kept at 150 °C for 1 h in an oven. Finally, after cooling to room temperature, the Ti fabric with the as-prepared samples was rinsed with deionized water and dried at 80 °C for 12 h.

### Synthesis of a BiVO_4_-rGO nanocomposite photocatalysts

First of all, graphene oxide (GO) was prepared by a modified Hummer’s method^30^, graphene oxide (0.057 g) was sonicated in 100 mL water for 10 min. The nanocomposites were prepared by mixing the prepared graphene oxide suspension into the solution with NH_4_VO_3_, oxalic acid, hexamethylenetetramine and Bi (NO_3_)_3_ 5H_2_O (the molar ratio 30:60:6:1), which was followed by vigorous magnetic stirring at room temperature for 1 h. Finally, the resulting mixture and a piece of pretreated Ti foil were then transferred to an autoclave and kept at 150 °C for 1 h. The final products were collected by centrifugation, and washed with deionized water and ethanol for three times, before drying at 80 °C for 12 h. The BiVO_4_-rGO nanocomposites obtained by annealing the as-prepared samples attached to the Ti foil substrate at 200 °C in nitrogen for 2 h with a heating rate of 1 °C min^−1^. To investigate the effect of GO concentration on the formation of BiVO_4_-rGO nanocomposites, GO solutions with different concentrations (0, 0.029, 0.057, 0.086 and 0.114 gL^−1^) were used in the same procedure, and are referred to as BiVO_4_-0, BiVO_4_-0.029, BiVO_4_-0.057, BiVO_4_-0.086 and BiVO_4_-0.114, respectively, while keeping other conditions unchanged.

### Photocatalytic Activity Measurements

Photocatalytic activities of the samples were evaluated by the degradation of methyl orange (MO) solution under simulated sunlight (λ ≥ 420 nm) in a homemade reactor with a cooling water circulator assembled to keep the reactor at a constant temperature. Experiments were performed at ambient temperature as follows: BiVO_4_ or BiVO_4_-rGO nanocomposites (2 cm × 2 cm) grown on Ti fabric catalyst was added into 50 mL of 10 mg/L methyl orange (MO) solution. Before illumination, the solution was stirred for 30 min in the dark in order to reach the adsorption-desorption equilibrium for MO and dissolved oxygen. A 300 W xenon lamp with a 420 nm cutoff filter to remove any irradiation below 420 nm was used as the visible light source to trigger the photocatalytic reaction. The concentrations of the MO were monitored using a UV-2003 UV-vis spectrophotometer by checking the absorbance at 464 nm during the photodegradation process. A sample in approximately 2 mL was taken at the designed time interval during irradiation for chromatographic analysis.

The photocurrent measurements had been taken on a electrochemical working station (CHI-660C, China). The active area of the specimen was 2 × 2 cm^2^ and the supporting electrolyte was 0.25 M Na_2_SO_4_ aqueous solution. A 300 W Xe-lamp was used to provide the simulated sunlight.

### Characterization

The samples were characterized with X-ray diffraction (XRD; Bruker D8 X-ray diffractometer). The morphology of the sample was investigated by a field-emission scanning electron microscope (FE-SEM; Hitachi S-4800) and a transmission electron microscope (TEM; JEOL-2100F at 200 kV). Energy Dispersive Spectroscopy (EDS) were used to determine morphology and elemental composition of the sample in the TEM. Raman spectra of GO, BiVO_4_, and BiVO_4_-rGO were recorded using a Raman spectroscope (JY-HR800, the excitation wavelength of 633 nm). UV-vis diffuse reflectance spectra (DRS) of the as-prepared samples were obtained using a Shimadzu UV-2550 spectrophotometer equipped with an integrating sphere using BaSO_4_ as the reflectance standard. The chemical composition of the sample was analyzed by X-ray photoelectron spectroscopy (XPS) using KAlpha 1063 (Thermo Fisher Scientific, UK). The photoluminescence (PL) spectral measurements were carried out on a Hitachi F-2500 fluorescence spectrophotometer with a Xe lamp as the light source.
