# Author Correction: BiVO_4_-rGO with a novel structure on steel fabric used as high-performance photocatalysts

**DOI:** 10.1038/s41598-020-62437-6

**Published:** 2020-03-24

**Authors:** Dong Fang, Xiujuan Li, Hui Liu, Weilin Xu, Ming Jiang, Wenbin Li, Xin Fan

**Affiliations:** 1 Key Lab of Green Processing and Functional Textiles of New Textile Materials Ministry of Education, College of Material Science and Engineering, Wuhan Textile University, Wuhan, 410000 P. R. China; 2 School of Metallurgy and Environment, Central South University, Changsha, 410083 P. R. China; 3 College of Materials Science and Engineering, Guilin University of Technology, Guilin, 541004 P. R. China

Correction to: *Scientific Reports* 10.1038/s41598-017-07342-1, published online 11 August 2017

This Article contains an incorrect version of Figure 1b. The correct Figure 1 appears below.

**Figure 1:**
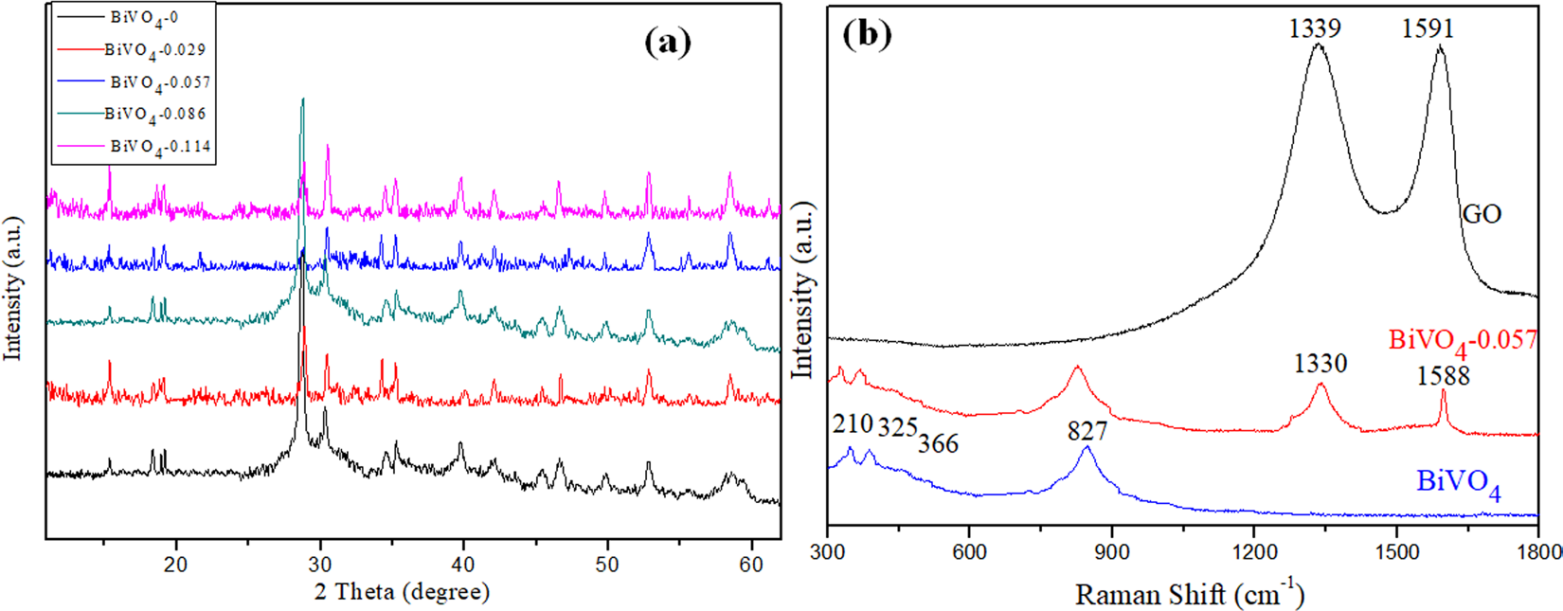
.

